# Development of Systematic Reviews to Inform WHO’s Recommendations for Elimination and Prevention of Re-Establishment of Malaria: Methodology

**DOI:** 10.4269/ajtmh.22-0740

**Published:** 2023-12-20

**Authors:** Maria Tusell, Laura C. Steinhardt, Julie Gutman, Zachary D. Schneider, Beena Bhamani, Monica P. Shah, Elisabet Martí Coma-Cros, John E. Gimnig, Koya C. Allen, Elie A. Akl, Kim A. Lindblade

**Affiliations:** ^1^ Barcelona Institute for Global Health (ISGlobal), Hospital Clínic – Universitat de Barcelona, Barcelona, Spain;; ^2^ Malaria Branch, Division of Parasitic Diseases and Malaria, Centers for Disease Control and Prevention, Atlanta, Georgia;; ^3^ Department of Internal Medicine, American University of Beirut, Beirut, Lebanon;; ^4^ Department of Health Research Methods, Evidence, and Impact, McMaster University, Hamilton, Canada;; ^5^ Global Malaria Programme, World Health Organization, Geneva, Switzerland

## Abstract

The basis for an evidence-based recommendation is a well-conducted systematic review that synthesizes the primary literature relevant to the policy or program question of interest. In 2020, the WHO commissioned 10 systematic reviews of potential interventions in elimination or post-elimination settings to summarize their impact on malaria transmission. This paper describes the general methods used to conduct this series of systematic reviews and notes where individual reviews diverged from the common methodology. The paper also presents lessons learned from conducting the systematic reviews to make similar future efforts more efficient, standardized, and streamlined.

## INTRODUCTION

Evidence-based guidelines for public health practice are important to ensure that the interventions and programs recommended for implementation are likely to be beneficial and acceptable to the target population, feasible to implement, and cost-effective and to avoid negatively impacting health equity. The basis for an evidence-based recommendation is a well-conducted systematic review that synthesizes the primary literature relevant to the policy or program question of interest. Systematic reviews contribute to the guideline development process by ensuring that all members of a guideline development group (GDG) consider an up-to-date and high-quality synthesis of the body of evidence that is relevant to the question at hand.^1^

In 2020, the WHO commissioned 10 systematic reviews of potential interventions in elimination or post-elimination settings to summarize their impact on malaria transmission outcomes and contextual factors that help determine their relevance for public health.^2^ The WHO’s elimination GDG considered the findings of these systematic reviews and developed 12 recommendations on malaria elimination published as part of the *WHO Guidelines for Malaria*.^3^

This paper describes the general methods used to conduct this series of systematic reviews and meta-analyses on interventions for malaria elimination and notes where individual reviews diverged from the common methodology. The paper also presents lessons learned to make similar future efforts more efficient, standardized, and streamlined.

## MATERIALS AND METHODS

### Information sources and search strategies.

We identified the studies included in this review by searching in relevant electronic databases and gray literature without time frame limitations. We used predefined search terms and relevant subheadings, as well as keywords related to malaria, antimalarials, diagnosis, and treatment. We included the following sources:

• Bibliographic databases: For all reviews, we searched MEDLINE (PubMED), EMBASE (Ovid), and the Cochrane Library. For the reviews of mass testing and treatment (MTaT)^4^ and targeted interventions (targeted drug administration [TDA],^5^ targeted testing and treatment [TTaT],^6^ and TTaT at points of entry),^7^ we additionally searched LILACS. For mass drug administration (MDA),^8^ mass relapse prevention (MRP),^9^ and reviews of reactive interventions,^10^^,^^11^ we also searched Global Health (OVID), CINAHL (EbscoHost), and Scopus.
• Other relevant databases: For all reviews, we searched the archives of the WHO, U.S. National Institutes of Health Ongoing Trials Register (ClinicalTrials.gov), the International Standard Randomised Controlled Trial Number (ISRCTN) registry, the WHO’s International Clinical Trials Registry Platform (WHO ICTRP), and the MESA Track database. In addition, for the reviews of MTaT and targeted interventions,^4^^–^^7^ we searched Z Electronic Tables of Contents (ZETOC) and the Armed Forces Pesticide Management Board. For the MDA^8^ and MRP reviews,^9^ we searched ProQuest Natural Science Collection and the Tropical Diseases Bulletin.
• Conference proceedings: For the reviews of MTaT and targeted interventions,^4^^–^^7^ we reviewed the Johns Hopkins Bloomberg School of Public Health Future of Malaria Research Symposium, Keystone/MESA Symposium, Women in Malaria Conference, Doctors Without Borders (MSF) Scientific Days 2021, Asia Pacific Malaria Elimination Network (APMEN) Journal Clubs (TechTalks), and the 6th Southern Africa Malaria Research Conference proceedings. We reviewed the Seventh Multilateral Initiative on Malaria Pan-African Malaria Conference for MDA,^8^ MRP,^9^ and reactive interventions.^10^^,^^11^
• Researchers and organizations within the field and the reference lists of studies identified and included in the review were reviewed for additional relevant studies.

For all the reviews, the search strategy was developed in collaboration with a systematic review information specialist. The primary searches for the MTaT and targeted interventions^4^^–^^7^ were conducted in March 2021 and updated in April 2022. For the reviews of MDA,^8^ MRP,^9^ and reactive interventions,^10^^,^^11^ searches were conducted in November 2020 and updated in March 2022 for reactive strategies and updated again in August 2022 for MDA^8^ and MRP.^9^ Specific search strategies for each individual review are presented in Supplemental Table 1 and with each report.^4^^–^^11^

### Eligibility criteria.

We included studies regardless of publication language or publication status (published, unpublished, in press, and in progress). Eligible study designs included the following:

• Randomized designs:
  ○ Cluster-randomized controlled trials (cRCTs) with at least two clusters per arm
  ○ Cluster-randomized cross-over trials with at least two clusters per arm
  ○ Cluster-randomized stepped wedge designs, with at least four clusters
  ○ Cluster-randomized factorial designs with at least two clusters per level
• Nonrandomized designs:
  ○ Quasi-experimental cluster-controlled trials with at least two clusters per arm
  ○ Controlled before and after studies with at least two clusters per arm and a contemporaneous control group
  ○ Interrupted time series studies (with or without a contemporaneous control group) with a clearly defined point in time when the intervention occurred; the studies should have had at least three data points before the start of the intervention and at least three data points after the intervention covering the same months as the pre-intervention data points and similar co-interventions before and after the start of the intervention
  ○ Uncontrolled before and after studies and before and after studies with historical controls (not applicable to all reviews).

For the review of interventions that conducted testing and treatment at points of entry,^7^ additional eligible study designs included prospective and retrospective cohorts and case-control and cross-sectional surveys.

We excluded randomized studies in the following situations: the follow-up periods for intervention and control arms were not the same; the timing of baseline and endline surveys was not the same in both the intervention and control arms; background interventions were different between the intervention and control arms; or co-interventions were implemented in one arm but not in the other. Interrupted time series studies were excluded if the pre- and post-intervention periods were different lengths or covered different seasons.

The eligible populations were specific to each review question and have been described elsewhere.^2^^,^^4^^–^^11^

### Study selection and data collection.

Two reviewers independently screened the titles and abstracts identified through the literature search using a checklist based on the eligibility criteria at the title and abstract stage. All studies assessed to be potentially eligible were retrieved, and the full reports were independently evaluated for eligibility by two reviewers using the full-text eligibility checklist. The two reviewers resolved any discrepancies through discussion or with the help of a third reviewer when needed. Prior to beginning full-text screening and data abstraction, a calibration exercise was conducted to ensure that there was good concordance among reviewers. After data extraction, the primary author, with confirmation by a second author, assessed the potential for each study to be included in the pooled analyses based on available outcome data and, if relevant, time points of follow-up.

### Outcomes related to benefits and harms.

Studies had to report at least one outcome among the outcomes of interest to be included in the review (see Table 1 for the definitions of each outcome). The GDG prioritized the list of outcomes for each intervention.^2^

**Table 1:** Definitions of the outcome measures

Outcome Measure	Definition
Measured at the population level	
Incidence of malaria infection	Number of malaria infections (diagnostically confirmed) identified through active surveillance divided by the product of the population (e.g., cohort) and the time under observation (person-time)
Prevalence of malaria infection	Number of people with malaria parasitemia (diagnostically confirmed) divided by the number tested, among a given population
Elimination	Zero indigenous or local cases for a period during the peak transmission season
Incidence of clinical malaria or number of indigenous malaria cases	Number of symptomatic local (indigenous) malaria cases among a certain population over a given time period; if measured by passive case detection, typically expressed as the number of cases divided by the population (for the time period specified); if measured in an incidence cohort, typically expressed as the number of cases divided by person-time
Drug resistance (MDA, MTaT, TDA, and TTaT reviews)	Presence of genetic changes (mutations or gene amplification) that confer reduced susceptibility to the drug
Insecticide resistance (Reactive Indoor Residual Spraying review)	Reduced susceptibility of mosquitoes to insecticide (e.g., low mosquito knockdown or mortality rates)
Proportion of imported infections identified at the border (TTaT at point of entry review)	Number of imported cases found at the border as a proportion of total imported cases
Among those receiving the intervention	
Adverse events	Known adverse events for the given antimalarial medication or insecticide used
Serious adverse events	Adverse events that result in death, requirement for or prolongation of inpatient hospitalization, or significant disability or incapacity or are life-threatening
Prevalence of infection	Number of people with malaria parasitemia (diagnostically confirmed) divided by the number tested, among the population (or a subset) receiving the intervention
Drug resistance	Presence of genetic changes (mutations or gene amplification) that confer reduced susceptibility to the drug

MDA = mass drug administration; MTaT = mass testing and treatment; TDA = targeted drug administration; TTaT = targeted testing and treatment.

As most of the interventions were evaluated based on their impact on malaria transmission toward elimination, primary outcomes (malaria incidence and prevalence) were measured at the population level rather than just in the group of individuals directly targeted by the intervention (Table 1). However, if population-level outcomes were not measured, the GDG also considered the impact of the intervention on the group of individuals directly targeted by the intervention as indirect evidence. Although some potential harms were relevant only to those targeted by the intervention (e.g., adverse events of medications), other harms were relevant at the population level (e.g., drug or insecticide resistance).

### Contextual factors and modeling.

The WHO considers contextual factors in developing recommendations to provide a health system’s perspective (Box 1). Although we did not conduct literature searches specifically for these factors, we systematically identified relevant articles when screening the results of our literature search. Information on five contextual factors^12^ was summarized for each intervention. For this, we included all primary study designs, including economic evaluations, qualitative studies, and other programmatic evaluations.

**Box 1:** Contextual factors^12^

**Values and preferences**: The relative importance assigned to health outcomes by those affected by them; how such importance varies within and across populations; and whether this importance or variability is surrounded by uncertainty **Acceptability**: The extent to which those benefiting from an intervention as well as other relevant stakeholder groups consider the intervention to be appropriate based on anticipated or experienced cognitive and emotional responses to the intervention **Health equity, equality, and nondiscrimination**: The extent to which the intervention benefits all populations; does not discriminate against anyone on the basis of sex, age, ethnicity, culture, language, sexual orientation or gender identity, disability status, education, socioeconomic status, residence, or any other characteristic; this could include whether the benefits and harms are equally distributed and whether the intervention is equally affordable and accessible to all **Financial and economic considerations**: The cost, overall economic impact, cost-benefit, and/or cost-effectiveness **Feasibility and health system considerations**: Barriers to implementation (resources available, programmatic considerations, existing and necessary infrastructure, etc.), interaction with the existing health system, and need for and use of the existing health workforce (including training)

In addition, we included all types of mathematical modeling studies, including designs using compartmental and/or individual-based models, and reporting results on how variation in operational parameters might affect outcomes.

### Data extraction and management.

Two reviewers independently extracted information from full-text articles using a pre-piloted paper or electronic data extraction form. Any discrepancies in information abstracted by the two reviewers were resolved by discussion to reach consensus or by consultation with a third reviewer. We contacted authors to ask for any information that was not reported in the study documents. For data that could not be retrieved, missing data were not imputed. Data extracted from studies included both descriptive data and adjusted analyses. Variables for data extraction included study design, setting, demographics, methodology, treatment, comparison, time period, baseline characteristics, intervention, outcomes, study biases as discussed by study authors, background interventions, and any other information on the study context that was informative in assessing the certainty of evidence.

### Risk of bias in individual studies.

Two members of the review team independently assessed the risk of bias for each study included in the review and for each specific outcome. Different tools to assess the risk of bias were used depending on the study design. For cRCTs, we used the Cochrane Risk of Bias Tool for randomized trials^13^^,^^14^ and assessed bias as low risk, some concerns, or high risk; results were displayed as summary graphs. For nonrandomized studies included in the reviews of MDA,^8^ MRP,^9^ and reactive interventions,^10^^,^^11^ we used a modification of criteria proposed by the Grading of Recommendations, Assessment, Development and Evaluations (GRADE) working group.^15^ For nonrandomized studies included in the reviews of MTaT and targeted interventions,^4^^–^^7^ we used the Risk of Bias Tool for nonrandomized studies for interventions from the Cochrane Effective Practice and Organization of Care Group.^16^ Any disagreements or discrepancies between reviewers were resolved through discussion or by consulting the other systematic review members.

### Synthesis of results.

For study designs with a control group, the effect of the intervention was assessed using odds ratios, risk ratios, or rate ratios depending on how the outcome was reported. Measures of variance (e.g., standard error, intercorrelation coefficient, or coefficient of variance) were included when reported. Both crude and adjusted measures of effect (e.g., risk ratios, odds ratio, rate ratios, or mean differences) were recorded with their 95% CIs. Data from randomized and nonrandomized studies were pooled separately.

We used the *I*^2^ statistic to assess the degree of heterogeneity in each meta-analysis. *I*^2^ represents the percentage of total variation across studies that is not due to chance. We interpreted heterogeneity according to a scale of very low, low, moderate, or high with values of <25%, 26–50%, 51–74%, and >75%, respectively.^17^ When there was substantial heterogeneity (*I*^2^ statistic value above 50% for the reviews of MTaT and targeted interventions^4^^–^^7^ and above 75% for the reviews of MDA,^8^ MRP,^9^ and reactive interventions)^10^^,^^11^ and/or inconsistency in the direction of the effect with non-overlapping CIs, then a meta-analysis was not performed.

We used fixed-effects meta-analysis to combine data when there was no substantial heterogeneity, only two studies, or data from multiple small but biased studies and one well-conducted study. Otherwise, we used random-effects meta-analysis. Studies using uncontrolled before and after study designs were not included in the meta-analyses. When there were enough studies, we conducted subgroup meta-analyses to explore potential effect modifiers identified by the GDG (Supplemental Table 2).^2^

### Grading of recommendations, assessment, development and evaluations.

For every intervention, we rated the certainty of the body of evidence for each outcome using the GRADE approach (Supplemental Table 3).^18^^,^^19^ Randomized studies were initially ranked as high certainty of evidence, and nonrandomized studies were initially ranked as low certainty of evidence. The certainty of evidence was rated down if there was evidence of the following: 1) risk of bias, 2) imprecision, 3) inconsistency, 4) indirectness, or 5) publication bias. Certainty of evidence for nonrandomized studies could be upgraded if all domains were rated as low risk and 1) there was evidence of a large magnitude of effect, 2) there was evidence of a dose-response relationship, or 3) when it was likely that residual confounding would increase the magnitude of the effect.

In addition, for the reviews of MTaT and targeted interventions,^4^^–^^7^ thresholds for determining the public health value of interventions were determined as listed in Supplemental Table 4 to ensure consistency in judgments around precision and size of absolute effect measures of interventions to reduce transmission.^20^ Therefore, the summary of findings tables in these reviews included both the relative and absolute sizes of effects and the certainty of evidence assessments (Supplemental Table 5).^21^

We narratively summarized the findings of studies on contextual factors. No assessment of the certainty of evidence for contextual factors was undertaken.

## CONCLUSION

One common finding across these 10 systematic reviews on malaria elimination is the paucity of rigorous studies. Fewer than six cRCTs were identified for any given review (four for MTaT, two for TTaT, two for TDA, zero for TTaT at point of entry, five for MDA, zero for MRP, five for reactive drug administration, three for reactive case detection and treatment, and two for reactive indoor residual spraying). The limited numbers of studies for each question precluded assessment of the potential effect modifiers selected a priori by the GDG. The only exception to this was the meta-analysis of MDA to reduce transmission of *Plasmodium falciparum*, which was stratified by level of transmission (very low to low and moderate to high).

With different teams conducting the various reviews, one best practice was the common use of a rigorous methodological approach; all reviews were based on the same core protocol but were adapted as needed. For example, for randomized studies, authors of the reviews of MTaT and targeted and reactive interventions used the adjusted effect sizes as analyzed by the study authors (when available), given the relatively small numbers of clusters in most trials and potential baseline imbalances, whereas the MDA reviews abstracted the effect sizes adjusted for clustering but unadjusted for other variables. In general, we attempted to include the “best” estimates of effect size, namely those adjusted for prespecified confounding factors, and included unadjusted analyses in footnotes as additional information, although in some cases unadjusted analyses were all that were able to be included.^22^ Likewise, decisions on information sources or agreed cutoffs for meta-analysis differed among reviews. To help standardize and streamline the process, future reviews could benefit by using a common protocol, especially when different teams and/or organizations are undertaking the systematic reviews.

One important lesson for future suites of reviews is the utility of prespecifying the thresholds for defining meaningful effect sizes for the interventions of interest and determining whether relative or absolute effect sizes are most relevant.^20^ This is best determined prior to beginning the systematic reviews through standardized approaches or by experts in the field, in this case the GDG. For example, what might be considered a small effect size for some malaria interventions might be considered a moderate or large effect size in low-transmission settings nearing elimination. Some reviews in this series used prespecified thresholds (e.g., the MDA,^8^ MTaT,^4^ and targeted reviews^5^^–^^7^) (see Supplemental Table 4). Future suites of reviews might benefit from standardizing these thresholds across reviews, with input from the GDG.^20^

Developing well-composed population, intervention, comparison, and outcome (PICO) questions is critical to ensure that the evidence included in the review is relevant to the question the GDG is interested in answering. For some reviews in this collection, the PICO questions as initially written were not specific enough to the settings that the GDG ultimately determined were most relevant. For example, even though the search originally captured studies in all transmission settings for TDA^5^ and TTaT,^6^ the GDG determined that those interventions would be most appropriate in very low– to low-transmission or post-elimination settings and modified the PICO questions accordingly. For TTaT at point of entry,^7^ the GDG noted that the PICO question could be interpreted to include two different forms of interventions: the traditional approach of TTaT at the time of entry through land crossings, seaports, or airports and the TTaT of organized or identifiable groups (e.g., military, migrant workers, or religious pilgrims) at some point in time after recently arriving or returning from malaria-endemic areas. Therefore, the GDG developed separate recommendations for these two interventions. To improve this process and ensure that all evidence included in the reviews is relevant, future suites of reviews could consider conducting a preliminary scoping exercise using a draft version of the PICO question. This could help to optimize the final version of the PICO question and improve the search and characterization of the interventions before the full literature searches are conducted.

To our knowledge, this is one of the first times that articles on contextual factors have been systematically identified and reviewed as part of the WHO malaria guideline development process.^12^ Relevant articles addressing contextual factors were identified when the results of each literature search were screened and were narratively summarized within each review. Notably, none of the reviews had meaningful contextual information on the values and preferences of persons affected by malaria with respect to the outcomes included in the review or on the impact of interventions on health equity, equality, and nondiscrimination. In future reviews, it might be useful for GDG members to specify which contextual factors are most salient and would contribute to and influence guideline development. For example, the values and preferences of persons affected by malaria for the outcomes evaluated might not be as salient a contextual factor for most malaria elimination interventions as they would be for other interventions, such as treatment options with various side effects for cancer patients. Many studies also collected information on population groups served by the interventions but did not explicitly evaluate the outcome by population groups, thus yielding very little data on health equity. In particular, this meant that none of the interventions could be evaluated for their impact on women’s health specifically.

It may be necessary to clearly acknowledge the possibility of minor changes in the reviews included in this *Supplement*, as they were used by the GDG in developing recommendations. Updates to the literature searches and some revisions have occurred, leading to versions that differ slightly from those considered by the GDG to develop its recommendations. However, these changes would not have impacted the outcomes. The original reviews have been published elsewhere.^23^^–^^31^

With the WHO’s Global Malaria Program expanding use of the quality-controlled guidelines development processes, we anticipate that similar suites of systematic reviews will continue to be conducted to develop the evidence base to inform the guidelines. To the extent that common methodological approaches can be used for such packages of reviews, the group developing the guidelines can more easily and systematically weigh recommendations across different interventions. Ideally, some of the approaches and lessons learned from conducting this suite of systematic reviews on malaria elimination interventions can help streamline the process for future systematic reviews used in the development of malaria guidelines.

## Supplemental Materials

10.4269/ajtmh.22-0740 Supplemental Materials
