# Supplementary material for: Development of Systematic Reviews to Inform WHO’s Recommendations for Elimination and Prevention of Re-Establishment of Malaria: Methodology
Source: Am J Trop Med Hyg. 2023 Dec 20;110(4 Suppl):11–6. doi: 10.4269/ajtmh.22-0740 (PMC10993789; doi:10.4269/ajtmh.22-0740)
Supplement: Supplemental Materials [file tpmd220740.SD1.pdf]

SUPPLEMENTAL DATA

Development of Systematic Reviews to Inform World Health Organization's Recommendations for Elimination and Prevention of Re-establishment of Malaria: Methodology

Maria Tusell,[1*±] Laura C. Steinhardt,[2±] Julie Gutman,[2] Zachary D. Schneider,[2] Beena Bhamani,[1] Monica P. Shah,[2] Elisabet Martí Coma-Cros,[1] John E. Gimnig,[2] Koya C. Allen,[1] Elie A. Akl,[3,4] Kimberly A. Lindblade[5]

[1] Barcelona Institute for Global Health (ISGlobal), Hospital Clínic - Universitat de Barcelona, Barcelona, Spain

[2] Malaria Branch, Division of Parasitic Diseases and Malaria, Centers for Disease Control and Prevention, Atlanta, GA USA

[3] Department of Internal Medicine, American University of Beirut, Beirut, Lebanon

[4] Department of Health Research Methods, Evidence, and Impact, McMaster University, Hamilton, ON, Canada

[5] Global Malaria Programme, World Health Organization, Geneva, Switzerland

[*] Address correspondence to Maria Tusell, Barcelona Institute for Global Health (ISGlobal), Hospital Clínic - Universitat de Barcelona, Carrer Rosselló 171 Entl. 2a, Barcelona 08036. Email: maria.tusell@isglobal.org
[±] These authors contributed equally.

Abbreviations

MDA	Mass drug administration
MRP	Mass relapse prevention
MTaT	Mass testing and treatment
RACDT	Reactive case detection and treatment
RDA	Reactive drug administration
RIRS	Reactive indoor residual spraying
TDA	Targeted drug administration
TTaT	Targeted testing and treatment
TTaT at POE	Targeted testing and treatment at points of entry

Supplemental Table 1. Information Sources and Search Terms

<table>
<tr><th colspan="4">INFORMATION SOURCES</th></tr>
<tr><th></th><th>Bibliographic databases</th><th>Other relevant databases</th><th>Conference proceedings</th></tr>
<tr><td>All reviews</td><td>MEDLINE (PubMED), EMBASE (Ovid), Cochrane Library</td><td>WHO Archives,
US National Institute of Health Ongoing Trials Register (clinicaltrials.gov),
ISRCTN registry,
WHO's International Clinical Trials Registry Platform (WHO ICTRP), MESA Track</td><td></td></tr>
<tr><td>MDA</td><td rowspan="2">Global Health (OVID), CINAHL (EbscoHost), Scopus</td><td rowspan="2">ProQuest Natural Science Collection, Tropical Diseases Bulletin</td><td rowspan="2">Seventh Multilateral Initiative on Malaria Pan-African Malaria Conference</td></tr>
<tr><td>MRP</td></tr>
<tr><td>MTaT</td><td rowspan="4">LILACS</td><td rowspan="4">ZETOC,
Armed Forces Pesticide Management Board</td><td rowspan="4">Johns Hopkins Bloomberg School of Public Health Future of Malaria Research Symposium,
Keystone/MESA Symposium, Women in Malaria Conference,
Doctors Without Borders Scientific Days 2021, Asia Pacific Malaria Elimination Network (APMEN) Journal Clubs (TechTalks),
6th Southern Africa Malaria Research Conference</td></tr>
<tr><td>TDA</td></tr>
<tr><td>TTaT</td></tr>
<tr><td>TTaT at PoE</td></tr>
<tr><td>RACDT</td><td rowspan="3">Global Health (OVID), CINAHL (EbscoHost), Scopus</td><td rowspan="3"></td><td rowspan="3">Seventh Multilateral Initiative on Malaria Pan-African Malaria Conference</td></tr>
<tr><td>RDA</td></tr>
<tr><td>RIRS</td></tr>
<tr><th colspan="4">SEARCH TERMS</th></tr>
<tr><td>MDA</td><td colspan="3">Malaria, antimalarials, mass chemoprophylaxis, mass drug administration, mass administration, mass screening and treatment, antimalarial agent</td></tr>
<tr><td>MRP</td><td colspan="3">[Same as MDA]</td></tr>
<tr><td>MTaT</td><td colspan="3">Malaria, antimalarials, falciparum, vivax, ovale, Plasmodium, malariae, mass test and treat, mass testing and treatment, MTAT, mass screening, screening, screened, diagnosed, diagnostics, test, mass, case detection, PACD, ACD, population-wide, community-wide, test, treat, screen, household screen, community case management, CCM, monthly screening and treatment, MSAT</td></tr>
</table>

**TDA**	Malaria, falciparum, vivax, ovale, Plasmodium, malariae, antimalarials, disease eradication, elimination, tailored intervention, treatment, strategy, administration, relapse prevention, presumptive treatment, therapy, focal drug administration, focal MDA, targeted intervention, treatment, strategy, administration, forest-goers, forests, vulnerable, worksites, farm, plantation, mining, miner, miners, laborers, cultivators, military, armed forces, military personnel, peace-keepers, agricultural, high-risk population, risk factors, special populations, high exposure, highly exposed, high transmission, hotspot
**TTaT**	Malaria, falciparum, vivax, ovale, Plasmodium, malariae, antimalarials, disease eradication, elimination, tailored intervention, treatment, strategy, administration, relapse prevention, presumptive treatment, therapy, focal drug administration, focal MDA, focal testing and treatment, FTAT, targeted intervention, treatment, strategy, administration, forest-goers, forests, vulnerable, worksites, farm, plantation, mining, miner, miners, laborers, cultivators, military, armed, forces, military personnel, peace-keepers, agricultural, high risk population, risk factors, special populations, high exposure, highly exposed, high transmission, hotspot, diagnostic techniques, procedures, screening, screened, diagnosed, diagnostics, test, point-of-care testing, rapid diagnostic test, RDT, parasitological diagnosis, parasitologic test, symptom screening, case detection, PACD, ACD
**TTaT at PoE**	Malaria, falciparum, vivax, ovale, Plasmodium, malariae, antimalarials, disease eradication, elimination, tailored intervention, treatment, strategy, administration, relapse prevention, presumptive treatment, therapy, focal drug administration, focal MDA, targeted intervention, treatment, strategy, administration, diagnostic techniques, procedures, screening, screened, diagnosed, diagnostics, test, point-of-care testing, rapid diagnostic test, RDT, parasitological diagnosis, parasitologic test, symptom screening, case detection, PACD, ACD, borders, frontier, immigration, immigrant, migrant, point of entry, cross-border, mobile population, returning worker, mobile worker, emigration, immigration, border crossing, travel, travel
**RACDT**	Malaria, screen, treat, test, treat, focal, foci, index, contact, reactive, detect, passive, administration, MDA
**RDA**	[Same as RACDT]
**RIRS**	Malaria, indoor, residual, spray, in-door, residual, pirimiphos-methyl, vector control, focal, IRS, reactive, index, close contact

Supplemental Table 2. Potential Effect Modifiers

MDA	Level of transmission<br>Vector control coverage<br>Malaria parasite species (*Pf, Pv, Po or Pm*) in the area<br>Antimalarial medication used, including gametocytocide or hypnozoiticide
MTaT	Level of transmission<br>Vector control coverage<br>Malaria parasite species (*Pf, Pv, Po or Pm*) of the index cases<br>Antimalarial medication used, including gametocytocide or hypnozoiticide<br>Whether or not screening for symptoms used before testing<br>Limit of detection and sensitivity of the test
MRP	Level of transmission<br>Vector control coverage<br>Medication used (Primaquine or Tafenoquine)<br>Use of G6PD testing to guide administration of medication<br>*P. vivax* strain (temperate vs. tropical)<br>Timing of the intervention (before vs. during transmission season)
TDA	Level of transmission<br>Vector control coverage<br>Malaria parasite species (*Pf, Pv, Po or Pm*) in the area<br>Antimalarial medication used, including gametocytocide and hypnozoiticide
TTaT	Level of transmission<br>Vector control coverage<br>Malaria parasite species (*Pf, Pv, Po or Pm*) in the area<br>Antimalarial medication used, including gametocytocide and hypnozoiticide<br>Whether or not screening for symptoms used before testing<br>Limit of detection and sensitivity of the test
TTaT at POE	Malaria parasite species (*Pf, Pv, Po or Pm*) in the area<br>Antimalarial medication used, including gametocytocide and hypnozoiticide<br>Low vs. high ratio of imported to indigenous cases<br>Limit of detection and sensitivity of the test
RACDT	Level of transmission<br>Vector control coverage<br>Malaria parasite species (*Pf, Pv, Po or Pm*) of the index cases<br>Antimalarial medication used, including gametocytocide or hypnozoiticide<br>Whether or not screening for symptoms used before testing<br>Limit of detection and sensitivity of the test<br>Size or population of intervention area around the confirmed case
RDA	Level of transmission<br>Vector control coverage<br>Malaria parasite species (*Pf, Pv, Po or Pm*) of the index cases<br>Antimalarial medication used, including gametocytocide or hypnozoiticide<br>Size or population of intervention area around the confirmed case
RIRS	Level of transmission<br>Vector control coverage<br>Malaria parasite species (*Pf, Pv, Po or Pm*) of the index case

	Size or population of intervention area around the confirmed case<br>Vector species

Supplemental Table 3. GRADE Criteria[1]

**Certainty**	**Interpretation**
Very low	The authors believe that the true effect is likely to be markedly different from the estimated effect
Low	The authors believe that the true effect might be markedly different from the estimated effect
Moderate	The authors believe that the true effect is probably close to the estimated effect
High	The authors have a lot of confidence that the true effect is similar to the estimated effect

Supplemental Table 4. Categorization of Effect Sizes

Species and transmission level	Effect size	Absolute difference in prevalence	Absolute difference in annual incidence
Plasmodium falciparum – Very low to low	Little to no	0 to 0.03% (0-0.3 per 1000)	0 to 3 per 1000 person-year
	Slight or some (public health value threshold)	0.04% to 0.1% (0.4 to 1 per 1000)	4 to 10 per 1000 person-year
	Large	>0.1% (>1 per 1000)	> 10 per 1000 person-year
Plasmodium falciparum – Moderate to high	Little to no	0 to 10% (0 to 100 per 1000)	0 to 250 per 1000 person-year
	Slight or some (public health value threshold)	10 to 20% (100 to 200 per 1000)	250 to 333 per 1000 person-year
	Large	>20% (>200 per 1000)	>333 per 1000 person-year
Plasmodium vivax	Little to no	0 to 5% (0 to 50 per 1000)	0 to 125 per 1000 person-year
	Slight or some (public health value threshold)	5% to 10% (50 to 100 per 1000)	125 to 250 per 1000 person-year
	Large	>10% (>100)	>250 per 1000 person-year

Supplemental Table 5. Summary of Findings Statements Criteria. Adapted from Santesso et al.[2]

	Certainty			
**Effect Size**	**High**	**Moderate**	**Low**	**Very Low**
**Large**	X results in a large reduction/ increase in outcome	X likely/probably results in a large reduction/increase in outcome	X may result in a large reduction/ increase in outcome	The evidence is very uncertain about the effect of X on outcome
**Sight or some**	X results in a slight reduction/ increase in outcome	X likely/ probably results in a slight reduction/ increase in outcome	X may reduce/increase outcome slightly	
**Little or no**	X results in little to no difference in outcome	X likely/ probably results in little to no difference in outcome	X may result in little to no difference in outcome	

References

1. Guyatt G, Oxman AD, Akl EA, Kunz R, Vist G, Brozek J, Norris S, Falck-Ytter Y, Glasziou P, DeBeer H, Jaeschke R, Rind D, Meerpohl J, Dahm P, Schünemann HJ., 2011. GRADE guidelines: 1. Introduction-GRADE evidence profiles and summary of findings tables. *J Clin Epidemiol 64*: 383–394
2. Santesso N, Glenton C, Dahm P, Garner P, Akl EA, Alper B, Brignardello-Petersen R, Carrasco-Labra A, De Beer H, Hultcrantz M, Kuijpers T, Meerpohl J, Morgan R, Mustafa R, Skoetz N, et al., 2020. GRADE guidelines 26: informative statements to communicate the findings of systematic reviews of interventions. *Journal of Clinical Epidemiology 119*: 126–135
